# Association between transmission rate and disease severity for *Actinobacillus pleuropneumoniae* infection in pigs

**DOI:** 10.1186/1297-9716-44-2

**Published:** 2013-01-11

**Authors:** Tijs J Tobias, Annemarie Bouma, Angeline JJM Daemen, Jaap A Wagenaar, Arjan Stegeman, Don Klinkenberg

**Affiliations:** 1 Department of Farm Animal Health, Faculty of Veterinary Medicine, Utrecht University, Yalelaan 7, Utrecht, 3584 CL, The Netherlands; 2 Department of Infectious Diseases and Immunology, Faculty of Veterinary Medicine, Utrecht University, Yalelaan 1, Utrecht, 3584 CL, The Netherlands; 3 Central Veterinary Institute of Wageningen UR, PO Box 65, Lelystad, 8200 AB, The Netherlands

## Abstract

A better understanding of the variation in infectivity and its relation with clinical signs may help to improve measures to control and prevent (clinical) outbreaks of diseases. Here we investigated the role of disease severity on infectivity and transmission of *Actinobacillus pleuropneumoniae*, a bacterium causing respiratory problems in pig farms. We carried out transmission experiments with 10 pairs of caesarean-derived, colostrum-deprived pigs. In each pair, one pig was inoculated intranasally with 5 × 10^6^ CFUs of *A. pleuropneumoniae* strain 1536 and housed together with a contact pig. Clinical signs were scored and the course of infection was observed by bacterial examination and qPCR analysis of tonsillar brush and nasal swab samples. In 6 out of 10 pairs transmission to contact pigs was observed, but disease scores in contact infected pigs were low compared to the score in inoculated pigs. Whereas disease score was positively associated with bacterial load in inoculated pigs and bacterial load with the transmission rate, the disease score had a negative association with transmission. These findings indicate that in pigs with equal bacterial load, those with higher clinical scores transmit *A. pleuropneumoniae* less efficiently. Finally, the correlation between disease score in inoculated pigs and in positive contact pigs was low. Although translation of experimental work towards farm level has limitations, our results suggest that clinical outbreaks of *A. pleuropneumoniae* are unlikely to be caused only by spread of the pathogen by clinically diseased pigs, but may rather be the result of development of clinical signs in already infected pigs.

## Introduction

The course of an infection can vary widely between individual hosts, affecting clinical signs of the infected host as well as the ability to transmit the infection to another member of the population. Variation in infectivity between individuals has shown to be of importance for the course of an epidemic [1-3] and can be associated with variation in clinical signs [4-6]. For some diseases, effective control measures should therefore target the severely diseased individual to reduce the size of an outbreak [7].

Current control measures for bacterial diseases in livestock farming often rely on an intervention with antimicrobials for the prevention or treatment of disease. As the use of antimicrobials is under debate, proper insight in the course of outbreaks and associations between disease and pathogen dispersion during outbreaks is crucial to design alternative control measures.

*A. pleuropneumoniae* is such a pathogen, causing clinical outbreaks and commonly controlled by antimicrobials, with high variation in infectivity [8]. Worldwide, *A. pleuropneumoniae* is highly prevalent among pig farms [9] and outbreaks of severe respiratory disease, with fibro hemorrhagic necrotizing pneumonia and fibrinous pleurisy [10], are observed occasionally. Variation in outbreaks with respect to size [11] and severity [12,13] are related to factors such as strain and serotype differences [14-16], immunity of the host [17,18] and co-infections [19,20].

Currently, the association between disease and pathogen dispersion and thereby the clinical course of *A. pleuropneumoniae* outbreaks is poorly understood. It could be that already subclinically infected pigs develop clinical signs at the same time because of the presence of a risk factor (trigger) that may be either infectious or non infectious [21]. Alternatively, it may be that an outbreak starts with only a few diseased pigs, which rapidly spread a clinical form of the infection on the farm, a hypothesis that is supported by a study that describes a rapid increase of prevalence during and after outbreaks [22]. The second hypothesis suggests a strong association between clinical signs and infectiousness, as well as a strong association between clinical signs of infected pigs within a transmission chain.

The aim of this study is to assess for *A. pleuropneumoniae*, the association between severity of clinical disease, infectiousness and transmission. In an experimental setting, the variation in disease between inoculated pigs was used to relate disease severity to the excreted bacterial load, the rate of transmission to a contact exposed pig and disease severity in that contact exposed pig.

## Material and methods

### Experimental design

To study *A. pleuropneumoniae* transmission, ten pair-wise transmission trials, split up in two separate and identically performed runs, were conducted. Per run twelve Caesarean-derived, colostrum-deprived (CD/CD) piglets (Landrace × York × Pietrain) were reared in incubators until 21 days of age as described before [23]. CD/CD pigs were used as a result of pilot studies that showed interference of *Pseudomonas spp*. and other *Pasteurellaceae spp.* for detection and quantification of *A. pleuropneumoniae* Colony Forming Units (CFUs) in samples obtained from inoculated SPF piglets (free of *A. pleuropneumoniae)*. At day 21 pigs were moved to an experimental unit (see Additional file 1: Figure S1) with eight pens, length × width: 1.1 × 1.1 meter, with 1/3 slatted floor and walls of 0.8 meter high, as in Dekker et al. [23]. The experimental unit was equipped with a laminar ventilation air flow. The room temperature was set constant at 25°C and air speed at 2.4 m/s.

Pigs were ear tagged and randomly assigned to pen 1, 2 or 3 for an eight day habituation period until inoculation. The habituation period was included for acclimatisation to the conditions in the experimental unit, to improve feed intake as well as to train the animals for clinical examination. Because trained pigs are likely to respond less stressfully to physical examinations, measurement errors were thus minimised.

After the habituation period pigs were randomly assigned a status and a pair number. Pairs consisted of I-pigs, that were inoculated, and C-pigs, that were contact exposed. Before inoculation, all C-pigs were moved to their designated pens (Additional file 1: Figure S1 and Table 1). The I-pigs were housed in pen 1 for inoculation and moved to their pen six hours after inoculation. Per run two S-pigs were assigned as sentinel and housed together in a separate pen to monitor indirect transmission of *A. pleuropneumoniae*.

**Table 1:** **Cumulative clinical disease score, necropsy and bacteriology results for *A. pleuropneumoniae *infection per pig**

**Pair**	**Pig status**	**Run/pen**	**Survival until day**	**Clinical score**		**Pathology**				
				**RHS4**	**RHS20**	**Pleurisy score**	**Pneumonia score**	**Bacteriology**		
								**Lung sample**	**Nose tonsil**	**Tonsil**
1	I	1/4	4	31.13	86.23	20.7	6.6	+	+	+
	C		21	3.10	5.11	0	0	ND	-	-
2	I	1/5	4	13.68	82.74	19.3	1.0	+	+	+
	C		21	2.48	6.72	0	0.26	-	-	-
3	I	1/6	2	66.88	93.38	33.9	33.2	+	+	+
	C		21	2.35	6.26	0	1.5	-	-	-
4	I	1/7	21	6.80	6.23	0	0	ND	-	+
	C		21	7.90	8.90	0	0	ND	-	+
5	I	1/8	21	16.18	12.87	5.9	0.3	+	+	+
	C		21	5.58	8.77	3.2	1.6	-	-	± dub
6	I	2/4	7	23.70	72.13	6.9	4.7	+	+	+
	C		21	7.43	6.42	0	0	ND	-	+
7	I	2/5	21	17.65	10.35	7.1	8.6	-	-	+
	C		21	8.70	5.55	0	0	ND	-	+
8	I	2/6	21	24.40	12.70	0	0	+	+	+
	C		21	4.03	4.20	0	0	ND	-	-
9	I	2/7	21	30.70	20.09	9.2	15.0	+	+	+
	C		21	7.13	7.55	0	0	ND	-	-
10	I	2/8	4	46.13	89.23	28.1	26.6	+	+	+
	C		21	5.60	7.82	0	0	ND	-	-
11	S	1/3	11^*^	0.31	ND	0	0	ND	-	-
	S		21	0.89	5.39	0	0	-	-	-
12	S	2/3	21	0.68	4.05	0	0	-	-	-
	S		21	0.56	5.27	0	0	-	-	-

+ = *A. pleuropneumoniae* confirmed, ± dub = dubious growth, ND = Not determined, * euthanized due to lameness.

Experiments were approved by the Animal experiments committee of Utrecht University (AEC) (approval number DEC2010.II.02.25). When pigs had body temperature of > 40°C or showed eminent signs of pain they were treated with Fentanyl (B. Braun Melsungen AG, Melsungen, Germany). Fentanyl is a potent analgesic, but does not bear anti-inflammatory capacities. Pigs were euthanized when the results of the daily welfare assessment exceeded the criteria accorded by the AEC.

### Inoculation

Inoculation was performed intranasally with a six hour culture of *A. pleuropneumoniae* reference strain 1536. *A. pleuropneumoniae* was cultured on Heart Infusion agar with 5% sheep erythrocytes and 0.2% β-NAD (Nicotinamide adenine dinucleotide) (HIS-V) overnight at 37°C and 5% CO_2_. The next day 1 colony was suspended in 1 mL of saline and 50 μL was plated on a new HIS-V plate and incubated. After six hours, the plate was washed with 5 mL of saline and diluted to 2.5 × 10^6^ CFUs/mL guided by optical density measurements. Before inoculation pigs were sedated with Azaperon (Stressnil®, Janssen Animal Health, Brussels, Belgium) and subsequently pigs were inoculated by dripping 1 mL of the inoculum in each nostril during inhalation (total 5 × 10^6^ CFUs). Before and after inoculation the concentration of the inoculum was determined by plating of serial dilution series. Inoculation dose and method of administration were chosen based on the results of two pilot studies where sufficient variation in disease severity was obtained, when 10^6^ Colony Forming Units (CFUs) *A. pleuropneumoniae* serotype 2 (strain 1536) was intranasally administered in CD/CD pigs.

### Samples

Before examination, restraining or sampling, all personnel had to change boots, coverall and gloves for each pen. To minimise the risk of transmission due to sampling or examination the contact pig was always sampled before the inoculated pig.

Tonsil and nasal samples were collected on post inoculation day −1, 1, 2, 4, 6, 8, 11, 13, 15, 18 and 21, or before euthanasia of severely diseased animals. Nasal swabs were obtained with a small cotton swab (Applimed SA, Italy) and tonsil scrapings were obtained by brushing the tonsils for 10 s with a soft toothbrush. On day −1 and day 21 the pigs were bled.

### Bacteriology

Tonsil brush samples were submerged in 10 mL and nose swabs in 1 mL saline and thoroughly mixed for 20 min before selective bacteriologic examination (SBE). Subsequently, tenfold serial dilution series were made of tonsil brush (10^-1^, 10^-2^, 10^-3^) and nose swab samples (10^0^, 10^-1^, 10^-2^). Per dilution 50 μL was plated on a selective agar plate with Clindamycin, Gentamicin, Vancomycin and Amphotericin (CGVA plate) [24] and incubated at 37°C and 5% CO_2_. *A. pleuropneumoniae* suspected colonies were counted after overnight incubation. Per sample two suspected colonies were confirmed as *A. pleuropneumoniae* when positively tested for satellite growth, Christie–Atkins–Munch–Petersen (CAMP) reaction, urease and mannitol fermentation. Bacterial counts were back calculated to whole sample constituents in CFUs. Additional analyses were performed with an apxIVA qPCR [25] (see Additional file 2).

### Clinical disease score

A clinical score (CS) was obtained for each pig daily from day −8 to day 21 post inoculation by the same examiner. CS was calculated as the average score (on a 0 – 4 scale) for eight different clinical parameters, scored as described by Hoeltig [26]: behaviour, locomotion score, vomiting, body temperature, respiratory breathing type, respiratory sounds, breathing frequency and coughing. Unlike in Hoeltig et al., cyanosis was not included, because it was never observed. Neither was feed intake, which could only be observed per pair. Clinical scores were obtained twice a day from day 0 – 5 and once a day thereafter, while bacterial samples were collected less frequently. To include all information of clinical observations, average clinical scores (AvgCS) for the days of sampling were derived by averaging all observed CS from one bacterial sampling moment to the next. For example on day 11 the AvgCS was calculated based on the CS of day 9 until 11. To calculate a cumulative respiratory health score at day 4 and 20 (RHS4, RHS20), like in Hoeltig’s study, CS was summed until day 4 or 20 and five points per day were added for each remaining day after an animal had died or was euthanized. Subsequently, the summed score was expressed as a percentage of the maximum obtainable score.

### Serology

Serology was applied to confirm the cause of clinical symptoms in I-pigs, as well as to detect possible false negative culture or qPCR results in C-pigs. Analysis of serology was performed at the Animal Health Service (Deventer, the Netherlands). Serum samples of day −1 and at euthanasia were analysed by Complement Fixation Test (CFT) titration [27] and a commercial App serotype 2 ELISA (Biovet, St Hyacinthe, Canada). CFT results > 80 in final sample or a distinct (> 0.2) increase of optical density were considered indicative for seroconversion and infection. Additionally, serum samples obtained at euthanasia were analyzed by an ApxIV ELISA (Idexx, Maine, USA). Serum samples of pigs that died before day 14 were not analysed by CFT or ApxIV ELISA, because no seroconversion was expected.

### Pathology

At necropsy, all pigs were examined by a veterinary pathologist (at the Veterinary Pathology Diagnostic Centre, Utrecht University). Tonsils were removed and homogenised and macroscopic lung and pleurisy lesions were assessed. A lesion score per lung was calculated, as described by Hannan et al. [28]. Lung lesions and tonsil homogenates were sampled by bacteriologic culture.

### Statistical analyses

The effects of time and pig status (I or C) on AvgCS and the log_10_ + 1 of CFUs count in tonsil samples after day 0 were evaluated using mixed effect models with pig number as random effect, to account for repeated observations. Spearman’s rank correlation test was used to investigate the correlation between ranks of cumulative clinical scores (RHS4 or RHS20), CFT titres and pathology scores in individual pigs.

To study the effect of disease severity (AvgCS) on transmission from the I-pig to the C-pig (transmission chain), three analyses were performed. First, correlation between AvgCS and log_10_ CFUs found in tonsil and nasal samples was tested by Spearman’s Partial rank correlation using pig number as controlled variable.

Second, the effect of disease severity and bacterial load on the rate of transmission was evaluated. The change from a negative to at least two consecutive positive samples in the contact pig was considered indicative for transmission. Presence or absence of transmission between two samplings (0/1) was used as response variable in a Generalized Linear Model with a binomial error distribution, a complementary log-log link function [8] and ln (time) between samplings as offset. AvgCS and log_10_ CFUs +1 counts in tonsil and nasal samples were evaluated as model terms and effect estimates are provided with 95% profile confidence intervals. A sensitivity analysis was carried out with respect to the use of SBE as indicator for infectiousness and transmission, by using qPCR results as an additional test (see Additional file 2). The qPCR may be more sensitive than SBE, but specificity for indicating infected and infectious pigs rather than non-viable bacteria may be lower.

Finally, the association between disease severity scores within the transmission chain was analysed. The correlation between CS (AvgCS) of the I-pig, from day of inoculation onwards, with CS (AvgCS) of the C-pig, from the first day of a positive culture and onwards, within pairs, was evaluated using Spearman’s Partial rank correlation analysis. Pair number was used as controlled variable.

Statistical analyses were performed using statistical software R version 2.11.1 [29] and additional packages pcor.R and lme4. Corrected Akaike Information Criterion for finite sample sizes (AICc) was used to select the models fitting the data best [30].

## Results

### Bacteriology results

**Figure 1:**
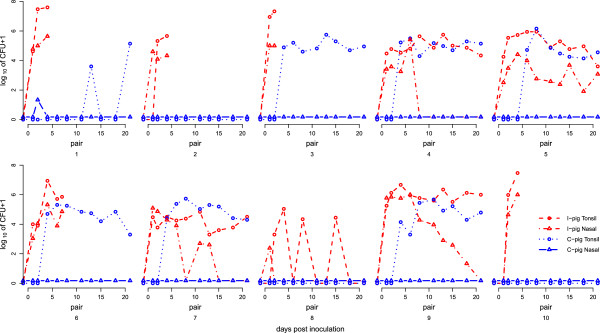
**Course of *A. pleuropneumoniae *CFUs counts in tonsil and nasal samples in time.** Tonsil sample counts are presented as *A. pleuropneumoniae* log_10_ (CFUs + 1) and nasal samples are presented as log_10_ (CFUs + 1.5) to separate them graphically from tonsil samples.

Inoculation of I-pigs was successful as demonstrated by at least two or more positive results in SBE in nasal or tonsillar samples in I-pigs upon inoculation. Sequentially taken tonsillar samples of I-pigs were almost all positive and more or less constant after day 2, results of nasal samples showed decreasing CFUs of *A. pleuropneumoniae* over time (Table 2 and Figure 1) and at day 21 only one of five surviving I-pigs had a positive nasal swab sample in SBE.

**Table 2:** **Results of tonsil and nasal sample selective bacterial examination for *A. pleuropneumoniae *in time**

**Pair**	**Status**	**Days post inoculation**											**Serology**			**Days to trans-mission based on SBE**
		**-1**	**1**	**2**	**4**	**6**	**8**	**11**	**13**	**15**	**18**	**21**	**CFT**	**App S2 ELISA**	**ApxIV ELISA**	
1	I	-	tn	tn	tn	†							ND	Neg	ND	
	C	-	-	tn	-	-	-	-	t	-	-	t	<40	Neg	Neg	∞
2	I	-	n	tn	tn	†							ND	Neg	ND	
	C	-	-	-	-	-	-	-	-	-	-	-	<40	Neg	Neg	∞
3	I	-	tn	tn	†								ND	Neg	ND	
	C	-	-	-	t	t	t	t	t	t	t	t	<40	Neg	Neg	4
4	I	-	tn	tn	tn	tn	t	t	t	t	t	t	640	Neg	Neg	
	C	-	-	-	t	t	t	t	t	t	t	t	<40	Neg	Neg	4
5	I	-	tn	tn	tn	tn	tn	tn	tn	tn	tn	tn	320	Neg	D	
	C	-	-	-	-	t	t	t	t	t	t	t	80	Neg	Neg	6
6	I	-	tn	tn	tn	tn	†						ND	Neg	ND	
	C	-	-	-	t	t	t	t	t	t	t	t	40	Neg	Neg	4
7	I	-	tn	tn	tn	tn	t	tn	tn	t	t	t	1280	Neg	P	
	C	-	-	-	t	t	t	t	t	t	t	t	<40	Neg	Neg	4
8	I	-	n	t	t	-	t	-	-	t	-	-	160	Neg	D	
	C	-	-	-	-	-	-	-	-	-	-	-	<40	Neg	Neg	∞
9	I	-	tn	tn	tn	tn	tn	tn	tn	tn	tn	t	1280	Neg	Neg	
	C	-	-	-	t	t	t	t	t	t	t	t	<40	Neg	Neg	4
10	I	-	n	tn	tn	†							ND	Neg	ND	
	C	-	-	-	-	-	-	-	-	-	-	-	<40	Neg	Neg	∞
																
11	S	-	-	-	-	-	-	†^*^					ND	Neg	ND	
	S	-	-	-	-	-	-	-	-	-	-	-	<40	Neg	Neg	
12	S	-	-	-	-	-	-	-	-	-	-	-	<40	Neg	Neg	
	S	-	-	-	-	-	-	-	-	-	-	-	<40	Neg	Neg	

Positive tonsil sample results are defined by t, positive nasal sample results are defined by n and negative samples by -. tn represents a positive tonsil and positive nasal sample result. † = euthanized or died before that day. * euthanized due to lameness. ND = not determined, Neg = Negative, D = Dubious, P = Positive.

Transmission occurred in six out of ten pairs as at least two or more positive samples in C-pigs tested positive (Figure 1). In one C-pig (pair 1) two non-consecutive samples were positive in culture, starting long after the I-pig had died and we assume that transmission did not occur in that pair. Only one nasal sample of the C-pigs showed a positive result in SBE (Figure 1).

Evaluation of mixed effect models showed that pig status significantly affected the CFUs count in tonsil samples. The median CFUs count in positive tonsil samples in I-pigs was 10^5.0^ (range: 10^3.3^ – 10^7.6^) CFUs and in transmission positive C-pigs: 10^4.9^ (range 10^3.3^ – 10^6.1^) CFUs. All tonsil and nasal samples from the S-pigs showed negative results in SBE.

Quantitative results from qPCR analysis and SBE were highly correlated (r = 0.89; *P* < 0.001 for all samples), as reported before [25]. As expected, more samples were tested positive with qPCR (204/454) than with SBE (153/454), though with low levels of DNA. Based on qPCR results, transmission occurred in eight pairs. Results and conclusions from qPCR analyses are presented in Additional file 2 and Additional file 3: Figure S2.

### Clinical disease

**Figure 2:**
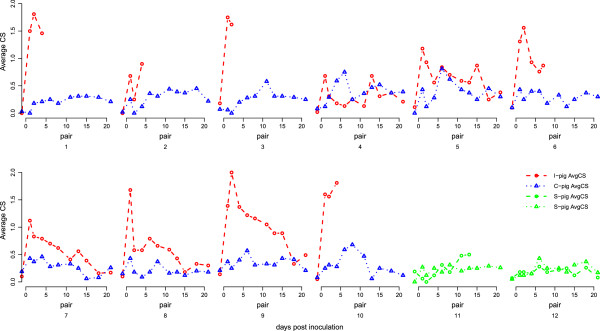
**Average Clinical Score (AvgCS) of piglets per pair in time.** The course of Average Clinical Score of piglets in time.

The CS in I-pigs varied between pigs ranging from 0 to 2.25 (median 0.66), see Table 3 and Figure 2. Clinical disease was more severe than expected from the pilot studies and five I-pigs died or were euthanized between days two and seven after inoculation. Each disease parameter under observation was affected at least once in I-pigs, except for cyanosis which was never observed in any of the pigs. Median CS was 0.3 (range 0.0 – 1.4) in all C-pigs and sentinel pigs (range 0.0 - 0.6). One of the sentinel pigs was euthanized on day 11 because of lameness, due to a sterile fissure in the right femur.

**Table 3:** Observed median and range of disease severity scores of the pigs

**Status**	**CS**	**AvgCS**	**RHS4**	**RHS20**
I-pigs	0.7 (0.0 – 2.3)	0.7 (0.1 – 2.0)	24.0 (6.8 – 66.9)	46.1 (6.3 – 93.4)
C-pigs +	0.3 (0.0 – 1.4)	0.3 (0.0 – 0.8)	7.3 (2.3 – 8.7)	34.9 (27.7 – 44.5)
C-pigs -	0.3 (0.0 – 0.9)	0.2 (0.0 – 0.7)	3.6 (2.5 – 5.6)	31.1 (21.0 – 33.6)
S-pig	0.3 (0.0 – 0.6)	0.2 (0.0 – 0.5)	3.1 (1.5 – 4.4)	5.3 (4.1 – 9.3)

I-pigs = inoculated pigs, C-pigs + = C-pigs in transmission positive pairs, C-pigs – = C-pigs in pairs with no observed transmission.

Statistical analyses demonstrated that CS and AvgCS were higher in I-pigs and *A. pleuropneumoniae* positive C-pigs compared to negative C-pigs or S-pigs. In addition CS and AvgCS were also significantly higher in I-pigs than in positive C-pigs. AvgCS decreased significantly over time in I-pigs, but not in positive C-pigs. Finally, *A. pleuropneumoniae* negative C-pigs and S-pigs did not differ in the score of CS nor AvgCS.

### Pathology

In 8 out of 10 I-pigs macroscopically observable lung or pleurisy lesions were observed (Table 1). Pleurisy and pneumonia scores were highly correlated within the same animal (Spearman’s rank correlation r = 0.89; *P* < 0.001). Three C-pigs had small pleurisy lesions and in one of them a small pneumonic lesion was observed. In sentinel pigs no pneumonia or pleurisy was observed. No associations were found between pathologic lesion scores of the pigs from the same pair.

Most tissue samples of macroscopic lung and pleurisy lesions were positive for *A. pleuropneumoniae*. No other bacteria were cultured from these lesions (Table 1). Tonsils of I-pigs were all positive for *A. pleuropneumoniae*, while not all positive C-pigs had a positive tonsil at necropsy.

### Serology

All 5 surviving I-pigs seroconverted in CFT (Table 1), with a median titre of 640. Nine C-pigs had negative CFT results and one positive C-pig had a dubious CFT result of 80. The two I-pigs that did not show any sign of pneumonia or pleurisy at necropsy had positive serum samples nevertheless. Results of apxIV ELISA were mostly in accordance with CFT results, except for the I-pigs of pair 4 and 9 (negative in ApxIV Elisa). With the App serotype 2 ELISA, none of the samples tested positive, nor did they show a distinct increase in OD.

### Association between clinical disease, pathology and serology results

Spearman’s correlation analyses showed significant correlations between cumulative RHS4 or RHS20 with pleurisy lesion scores (r = 0.77; *P* < 0.001, respectively r = 0.87 *P* < 0.001) and pneumonia scores (r > 0.59; *P* < 0.01, respectively r = 0.75 *P* < 0.001). Comparable results were found for the association between CFT results and pleurisies (r = 0.53; *P* = 0.04) and pneumonia (r = 0.75; *P* = 0.001) scores.

### Disease severity and bacterial load

**Figure 3:**
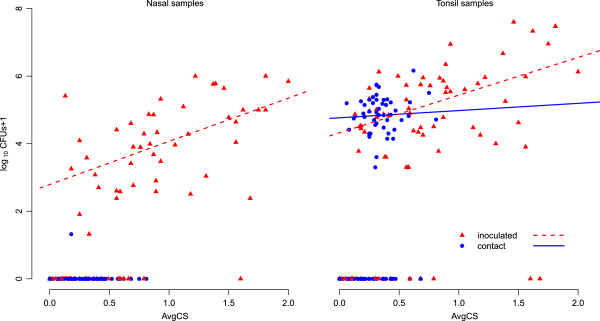
**Association between bacterial load and average disease score.** Association between log_10_ (CFUs +1) of *A. pleuropneumoniae* 1536 in nasal and tonsil samples and average clinical score (AvgCS) for I- and C-pigs. Regression lines are based on positive samples.

Partial correlation analysis showed that AvgCS was significantly correlated with bacterial quantities in tonsil (r = 0.54, *P* < 0.001) and nasal samples (r = 0.63 (*P* < 0.001) of I pigs, but not in tonsil samples of C-pigs (r = 0.06, *P* = 0.69) (Figure 3). This means that 29% and 39% of the variance in AvgCS in I-pigs could be explained by the variance in number of *A. pleuropneumoniae* found in tonsil or nasal samples respectively. Since only one positive nasal swab was found in C-pigs, no associations were made to AvgCS. Evaluation of mixed model with time as fixed effect and pig number as random effect, showed that pig status (I- or positive sampled C-pigs) is not needed to explain the height of the bacterial tonsil load by AvgCS. This means that bacterial tonsil load is equally affected by AvgCS in I-pigs and transmission positive C-pigs.

### Effects on transmission rate

Transmission was either observed on day 4 or 6 or not at all. The best fitting model (lowest AICc), evaluating the effects on the transmission rate, included both the log_10_ + 1 of nasal CFUs and average clinical score (Table 4). Based on the confidence intervals transmission was, negatively affected by AvgCS and positively by nasal bacterial load. Both effects are much smaller and not significant if included in the model individually. The results were not sensitive to the use of SBE rather than qPCR to define transmission and infectiousness (Additional file 2 and Additional file 4).

**Table 4:** **Effects of disease severity and bacterial counts on the transmission rate of *A. pleuropneumoniae *1536**

**Model**	**Intercept**	**Log**_**10**_**(CFUs + 1) Tonsil**	**Log**_**10**_**(CFUs + 1) Nasal**	**AvgCS**	**AICc**
1	−2.3 (−3.3; -1.5)	X	X	X	28.10
2	−4.0 (−7.8; -1.9)	0.35 (−0.04; 0.93)	X	X	27.39
3	−3.9 (−7.7; -2.0)	X	0.44 (−0.01; 1.17)	X	26.76
4	−2.1 (−4.0; -0.6)	X	X	−0.22 (−2.19; 1.40)	30.27
5	−4.1 (−8.0; -2.0)	0.11 (−0.44; 0.91)	0.34 (−0.36; 1.27)	X	29.01
6	−4.8 (−11.3; -1.6)	0.82 (0.10; 2.24)	X	−1.95 (−5.08; 0.16)	26.93
7	−4.3 (−11.5; -1.4)	X	1.07 (0.25; 3.06)	−2.48 (−5.80; -0.16)	24. 69
8	−5.0 (−12.6; -1.5)	0.31 (−0.21; 1.67)	0.88 (−0.10; 2.92)	−2.67 (−5.95; -0.30)	26.60

Model evaluation for estimation of the effects on the transmission rate, using a Generalized Linear Model with complementary log-log link. Effect estimators are given with the 95% confidence interval.

### Association of disease severity within pairs

In transmission positive pairs a significant association of CS between the I- and C-pig was found, r = 0.34 (*P* = 0.00). Based on AvgCS, r = 0.35 (*P* = 0.06).

## Discussion

In this study we investigated the association between transmission of *A. pleuropneumoniae*, bacterial load in the oropharynx and nasal cavity and severity of clinical signs. An association was to be expected if clinical outbreaks are caused by rapid spread of the bacteria by clinically affected pigs. The average clinical score was positively associated with the bacterial load - the amount of bacteria - in the oropharynx and nasal cavity which was, in turn, positively associated with the transmission rate of *A. pleuropneumoniae*. However, corrected for bacterial load, the clinical score was negatively associated with the transmission rate. This means that in pigs with a similar bacterial load, pigs with higher clinical scores transmitted the bacteria less efficiently. Both effects of clinical disease (positive and negative) resulted in a much smaller net effect, not even significantly from zero (Table 4, model 4). The association found between daily clinical score within the transmission chain was r = 0.34. These findings suggest that it is unlikely that clinical outbreaks of *A. pleuropneumoniae* are caused by rapid transmission of *A. pleuropneumoniae* by clinically affected pigs only. It implies that other causes and mechanisms may cause the occurrence of outbreaks.

Our conclusions were based on the results of bacterial examination, because SBE detects viable bacteria and is, to our opinion, representative for colonization. Additional analyses performed with qPCR (Additional file 2) suggested that transmission may have occurred in two additional pairs, but the number of genomic copies found in those C-pigs was low. Although sensitivity in the SBE is not 100%, we considered these samples containing non-viable bacteria and therefore concluded that these pigs were not infected. Therefore we included the qPCR results as additional data only. Most importantly however, the analysis of transmission based on the qPCR results would lead to the same conclusion about the effect of disease severity and nasal bacterial load on transmission.

Transmission of pathogens is dependent on the susceptibility and the infectiousness of the uninfected and infected individuals, respectively and the contact rate [31]. CD/CD pigs were used and randomly assigned to pairs and treatment. Therefore susceptibility of the pigs was considered to be similar at the start of the experiment. Severity of disease was positively associated with the bacterial load, and bacterial load with the transmission rate, but the severity of disease was negatively associated with the transmission rate. This result could be explained by the effect of disease severity on the contact rate, as *A. pleuropneumoniae* is assumed to spread mainly by direct contact. If clinical signs affect the frequency and/or intensity of contact between pigs, the rate of transmission may be lower when pigs are showing severe signs compared to sub-clinically infected pigs. On the other hand, in similarly affected animals the transmission rate will be mostly associated with the number of bacteria isolated, as has been shown by others [8].

An association between clinical score in I-pigs and positive C-pigs was demonstrated, but the score of inoculated pigs was significantly higher than for contact positive ones. This could be explained by the absence of lung lesions in five of six positive contact pigs. In positive C-pigs higher clinical scores were observed than in negative C-pigs, which was not reported in other studies [8,32]. This may be explained as follows. First, *A. pleuropneumoniae* might have caused only minor pathology in pig tonsils, as previously described for gnotobiotic pigs [33], which may have induced only mild clinical signs. We did not, however, investigate the morphologic changes in pig tonsils. Second, the scoring method we used was more detailed than in the other transmission studies and as signs were only mild a less detailed method might have missed these. It should be mentioned, however, that our scoring method was not applied blindly as the observer was aware of the infection status of each pig, which may have resulted in observation bias.

In previously performed studies with *A. pleuropneumoniae*, infectious pigs were induced by endobronchial application of the inoculum or by exposure to other infected pigs [24], resulting in a more uniform expression of clinical signs. In our study, the inoculum was applied intranasally, as we aimed at inducing variation in clinical signs rather than uniformity. It is known that variation in signs can also be affected by using different doses or different inoculation routes [15,34], but using these methods of challenge was not suitable here as a possible observed difference in transmission could then also be due to inoculation method rather than the clinical score.

In our study bacterial counts in tonsil and nasal sample from inoculated pigs correlated well, but the number of bacteria in nasal samples decreased over time and most nasal samples were found negative in contact infected pigs. Our findings suggest that for transmission studies on farms it is more appropriate to take tonsil samples instead of nasal samples to detect a colonised pig, as the nasal samples may be negative in pigs with low number of bacteria. In experimental studies nasal samples may be useful, as they are easier to collect and, moreover, may reflect the infectiousness of the pigs more appropriately [8].

In this study CD/CD pigs were used, because of diagnostic limitations of bacterial examination in SPF pigs. In the field specific maternally derived antibodies [18], and possibly cross immunity for other *Pasteurellaceae*, can be protective for developing clinical signs. Besides, competition for colonization by other bacteria, especially other *Pasteurellaceae*, or protective effects of milk or colostrum on colonization are plausible under field conditions as well. Therefore extrapolation of our estimates to the field should be done with extreme caution and our conclusion on the relation between clinical signs and transmission of *A. pleuropneumoniae* needs confirmation under more natural circumstances.

Nevertheless our observation that clinical signs reduce transmission of *A. pleuropneumoniae*, e.g. by reducing the contact rate, could have significant consequences for effectiveness of interventions. Isolation of severely affected individuals has been shown to be effective to reduce the size of an outbreak, e.g. for *Salmonella*[7] in cattle. In that case the development of clinical signs usually coincides with a raise in infectiousness and diseased animals are responsible for most of the transmission during the outbreak. The latter was exactly one of the hypotheses for the course of outbreaks of *A. pleuropneumoniae*. If they become diseased, *A. pleuropneumoniae* infected pigs become diseased within a few days after infection and this study has shown that diseased pigs shed more bacteria, so isolation theoretically might be effective. However, most infected pigs do not become diseased, so most infectious pigs will not be noticed. Moreover the results of this study have shown a negative impact of disease on transmission. Thus, while during an outbreak of pleuropneumonia, caused by *A. pleuropneumoniae*, isolation of severely affected pigs may be beneficial for their wellbeing, the effect on the course of the outbreak is likely to be limited.

In conclusion the results of this experiment do not support the hypothesis that outbreaks start with only a few diseased pigs that rapidly spread a clinical form of the infection. It is therefore more likely that an outbreak occurs due to the development of clinical signs at the same time in already infected pigs due to some (non-) infectious trigger.

## Competing interests

The authors declare that they do not have any competing interests.

## Authors’ contributions

TJT performed the animal experiment, was responsible for laboratory analyses, retrieving results and drafting and finalizing the manuscript. DK designed the study and assisted in statistical analysis and preparation of the manuscript. AB was involved in design of the study, laboratory analyses and preparation of the manuscript. AJJMD assisted with laboratory analyses and preparation of the inoculum. JAW and JAS were involved in design of the study and preparation and drafting the manuscript. All authors read and approved the final manuscript.

## Supplementary Material

Additional file 1: Figure S1 Spatial setup of the transmission experiment. For day −8 to 0 all pigs were randomly housed per 4 in pen 1 to 3. On day 0 all C-pigs were moved to their designated pen. All I-pigs were moved temporarily to pen 1 for inoculation. After 6 hours they were moved to their designated pen. From day 0.5 to day 21 all I and C pigs were only housed in pen 4 to 8; Sentinels were housed in pen 3. Click here for file

Additional file 2: Supplementary material 1 Additional analyses by qPCR. A detailed description is given on apxIVA qPCR analysis, results and interpretation. Furthermore results of statistical models for evaluation of transmission that include qPCR results are presented. Click here for file

Additional file 3: Figure S2 Results of qPCR analysis per pair in time. For day −1 to day 21 qPCR results are given in the number of genomic copies (g.c.) per sample. Tonsil sample amounts are presented as *A. pleuropneumoniae* log_10_ (g.c. + 1) and nasal samples are presented as log_10_ (g.c. + 1.5) to separate the results graphically from tonsil samples. Click here for file

Additional file 4: Supplementary material 2 Table S1 Effects of disease severity and genomic copies on the transmission rate of *A. pleuropneumoniae* 1536. Model evaluation for estimation of the effects on the transmission rate, using a Generalized Linear Model with complementary log-log link. Effect estimators are given with the 95% confidence interval. Click here for file
