# Supplementary figures and images for: Association between transmission rate and disease severity for Actinobacillus pleuropneumoniae infection in pigs
Source: Vet Res. 2013 Jan 11;44(1):2. doi: 10.1186/1297-9716-44-2 (PMC3574036; doi:10.1186/1297-9716-44-2)

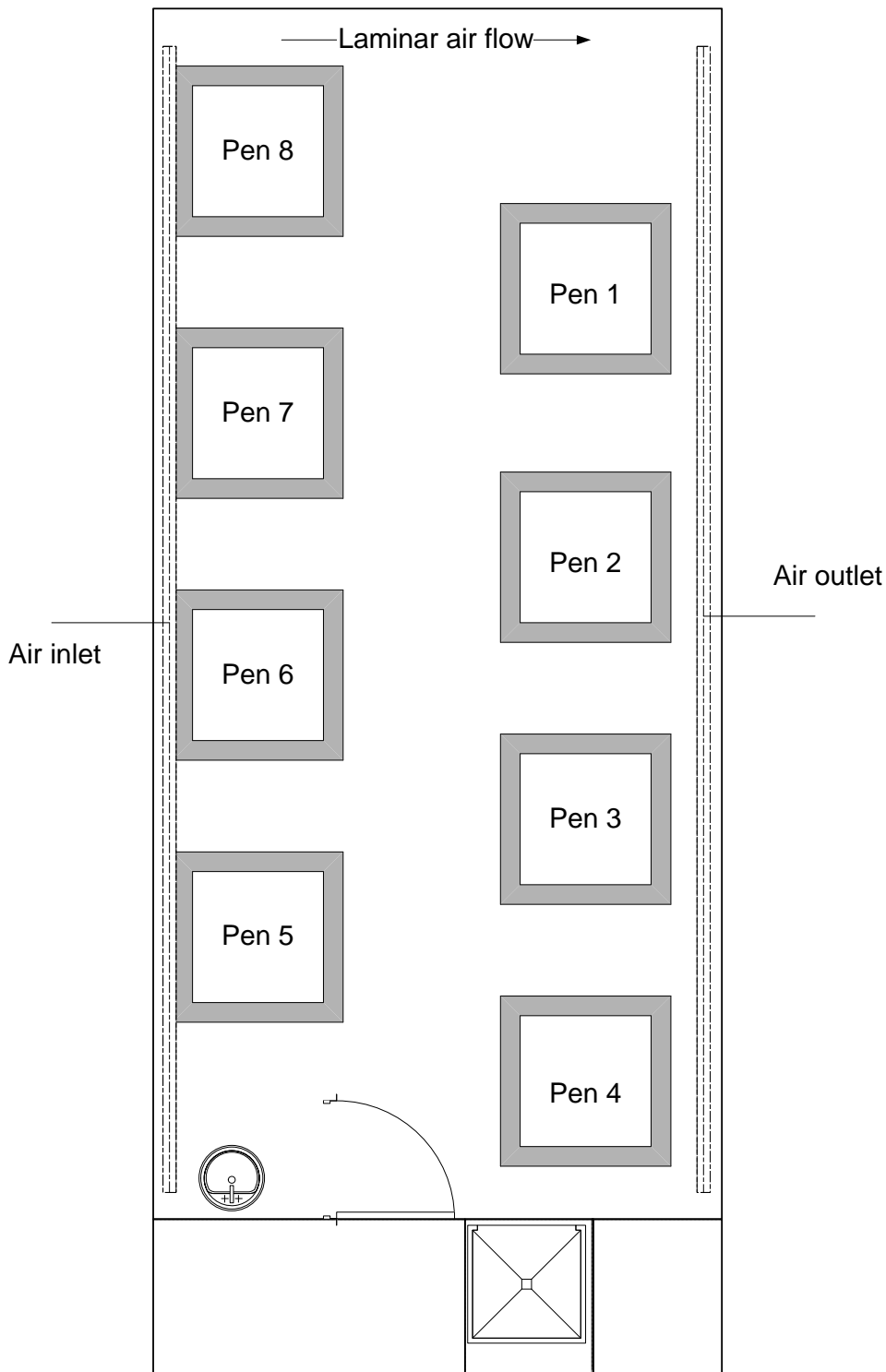

Supplement: Additional file 1: Figure S1 — Spatial setup of the transmission experiment. For day −8 to 0 all pigs were randomly housed per 4 in pen 1 to 3. On day 0 all C-pigs were moved to their designated pen. All I-pigs were moved temporarily to pen 1 for inoculation. After 6 hours they were moved to their designated pen. From day 0.5 to day 21 all I and C pigs were only housed in pen 4 to 8; Sentinels were housed in pen 3. [file 1297-9716-44-2-S1.pdf]

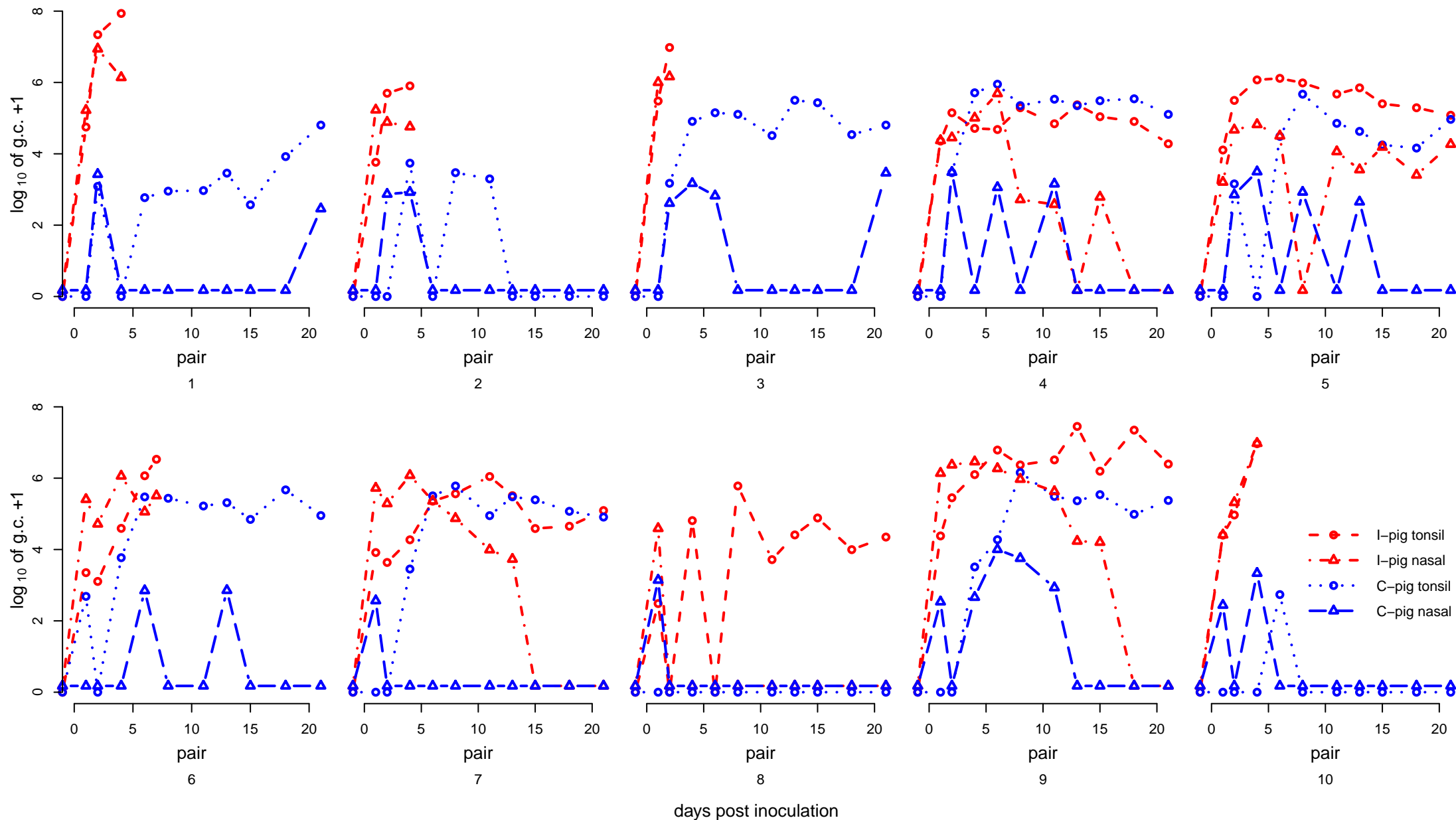

Supplement: Additional file 3: Figure S2 — Results of qPCR analysis per pair in time. For day −1 to day 21 qPCR results are given in the number of genomic copies (g.c.) per sample. Tonsil sample amounts are presented as A. pleuropneumoniae log10 (g.c. + 1) and nasal samples are presented as log10 (g.c. + 1.5) to separate the results graphically from tonsil samples. [file 1297-9716-44-2-S3.pdf]
