# Supplementary material for: Association between transmission rate and disease severity for Actinobacillus pleuropneumoniae infection in pigs
Source: Vet Res. 2013 Jan 11;44(1):2. doi: 10.1186/1297-9716-44-2 (PMC3574036; doi:10.1186/1297-9716-44-2)
Supplement: Additional file 2: Supplementary material 1 — Additional analyses by qPCR. A detailed description is given on apxIVA qPCR analysis, results and interpretation. Furthermore results of statistical models for evaluation of transmission that include qPCR results are presented. [file 1297-9716-44-2-S2.doc]

# Supplementary material 1

# Additional analyses based on qPCR

# Material and methods

In addition to analysis by SBE, all nasal and tonsil samples were analysed by qPCR, as described previously [25]. After retrieving material for SBE and DNA isolation (by use of Instagene Matrix (BioRad)), qPCR analysis was performed on a BioRad iQ5 thermocycler, and the number of genomic copies (g.c.) of *A. pleuropneumoniae* DNA in the initial sample, denoted on a Log_10_ scale, was obtained.

For statistical analysis of transmission, in the main text an animal was defined infected and infectious on a particular day when either nasal or tonsillar SBE was positive. Because SBE detects viable bacteria, it is very specific for detecting infectiousness, but sensitivity may not be optimal. Therefore, as a sensitivity analysis we re-analysed the data using qPCR as an additional test. To define a transmission event, we used two subsequent positive samples in either qPCR or SBE of the tonsil sample (as in the main text with only SBE). As explanatory variables for infectiousness we used the qPCR results on the nasal and tonsillar swabs of the infectious pig. According to these rules, the transmission events in two pairs (pairs 1 and 2) occurred shortly after removal of the infectious pig: one and two sampling events later, respectively. For these three sampling events without presence of the infectious pig, the last observations on the infectious pigs were used as explanatory variables.

# Results

The qPCR results are shown in Additional file 3: Supplementary figure 2. As expected, more samples were positive than in SBE, possibly due to higher sensitivity or lower specificity. Because qPCR results include DNA of non-viable bacteria as well, it was chosen to report transmission based on SBE results in the manuscript only.

The major difference of the qPCR results with the SBE results, is that the qPCR results indicate two more transmission events: in pairs 1 and 2. However, the amount of DNA in the samples was low.

Nevertheless, we analysed the data based on these new definitions and obtained the results in Additional file 4: Supplementary material 2. Basically, the results are very similar to the results of the original analysis. It is the same model that fitted the data best (lowest AICc), containing AvgCS and the nasal sample bacterial load (based on qPCR results). As in the original analysis, the direction of the effect estimator of AvgCS is below zero.
