# Supplementary material for: Association between transmission rate and disease severity for Actinobacillus pleuropneumoniae infection in pigs
Source: Vet Res. 2013 Jan 11;44(1):2. doi: 10.1186/1297-9716-44-2 (PMC3574036; doi:10.1186/1297-9716-44-2)
Supplement: Additional file 4: Supplementary material 2 Table S1 — Effects of disease severity and genomic copies on the transmission rate of A. pleuropneumoniae 1536. Model evaluation for estimation of the effects on the transmission rate, using a Generalized Linear Model with complementary log-log link. Effect estimators are given with the 95% confidence interval. [file 1297-9716-44-2-S4.doc]

# Supplementary Material 2

###### Supplementary Table 1 Effects of disease severity and genomic copies on the transmission rate of *A. pleuropneumoniae* 1536.

Model evaluation for estimation of the effects on the transmission rate, using a Generalized Linear Model with complementary log-log link. Effect estimators are given with the 95% confidence interval. Effect estimators are given with the 95% confidence interval.

| Model | Intercept | Log_10_ (g.c.+1) Tonsil | Log_10_ (g.c.+1) Nasal | AvgCS | AICc |
| --- | --- | --- | --- | --- | --- |
| 1 | -1.86 (-2.6; -1.2) | x | x | x | 39.53 |
| 2 | -4.28 (-7.6; -1.7) | 0.45 (-0.01; 0.97) | x | x | 38.20 |
| 3 | -4.24 (-8.1; -2.11) | x | 0.51 (0.11; 1.51) | x | 34.50 |
| 4 | -2.29 (-4.0; -0.9) | x | x | 0.46 (-0.92; 1.77) | 41.30 |
| 5 | -4.54 (-8.5; 1.8) | 0.10 (-0.44; 0.70) | 0.46 (0.0; 1.18) | x | 36.73 |
| 6 | -4.34 (-7.8; -1.6) | 0.50 (-0.04; 1.21) | x | -0.22 (-2.03; 1.33) | 40.49 |
| 7 | -5.33 (-12.1; -1.9) | x | 1.14 (0.33; 2.68) | -2.07 (-4.50; -0.06) | 32.77 |
| 8 | -6.31 (-14.3; -1.9) | 0.24 (-0.37; 0.94) | 1.10 (0.26; 2.7) | -2.29 (-4.94; -0.17) | 34.70 |
